# Emerging Evidence on the Effects of Dietary Factors on the Gut Microbiome in Colorectal Cancer

**DOI:** 10.3389/fnut.2021.718389

**Published:** 2021-10-11

**Authors:** Sandeep Appunni, Muni Rubens, Venkataraghavan Ramamoorthy, Raees Tonse, Anshul Saxena, Peter McGranaghan, Adeel Kaiser, Rupesh Kotecha

**Affiliations:** ^1^Government Medical College, Kozhikode, India; ^2^Department of Biochemistry, All India Institute of Medical Sciences, New Delhi, India; ^3^Office of Clinical Research, Miami Cancer Institute, Baptist Health South Florida, Miami, FL, United States; ^4^Baptist Health South Florida, Miami, FL, United States; ^5^Department of Radiation Oncology, Miami Cancer Institute, Baptist Health South Florida, Miami, FL, United States; ^6^Department of Radiation Oncology, Florida International University, Miami, FL, United States

**Keywords:** dietary fibers, short chain fatty acid, gut microbiota, colorectal cancer prevention, epigenetics

## Abstract

Dietary factors have important role in modulating the gut microbiome, which in-turn regulates the molecular events in colonic mucosa. The composition and resulting metabolism of the gut microbiome are decisive factors in colorectal cancer (CRC) tumorigenesis. Altered gut microbiome is associated with impaired immune response, and the release of carcinogenic or genotoxic substances which are the major microbiome-induced mechanisms implicated in CRC pathogenesis. Diets low in dietary fibers and phytomolecules as well as high in red meat are important dietary changes which predispose to CRC. Dietary fibers which reach the colon in an undigested form are further metabolized by the gut microbiome into enterocyte friendly metabolites such as short chain fatty acid (SCFA) which provide anti-inflammatory and anti-proliferative effects. Healthy microbiome supported by dietary fibers and phytomolecules could decrease cell proliferation by regulating the epigenetic events which activate proto-oncogenes and oncogenic pathways. Emerging evidence show that predominance of microbes such as *Fusobacterium nucleatum* can predispose the colonic mucosa to malignant transformation. Dietary and lifestyle modifications have been demonstrated to restrict the growth of potentially harmful opportunistic organisms. Synbiotics can protect the intestinal mucosa by improving immune response and decreasing the production of toxic metabolites, oxidative stress and cell proliferation. In this narrative review, we aim to update the emerging evidence on how diet could modulate the gut microbial composition and revive colonic epithelium. This review highlights the importance of healthy plant-based diet and related supplements in CRC prevention by improving the gut microbiome.

## Introduction

The gut microbiome includes the collective genes and genome of all microorganisms residing in the gastrointestinal tract (GIT) (1). There are over 100 trillion microbes residing in the human GIT, and majority of them are located in the colon (2). Metagenomic studies demonstrate that there are ~1,952 uncultured bacterial species in the human gut, and many remain unclassified to date. This contributes to substantial diversity within the microbial ecosystem (3). The host-microbe relationship can be symbiotic or pathogenic. Several external factors, such as diet, medication, and lifestyle heavily influence the microbial ecosystem (4). Symbiotic relationships between host and microbes have a plethora of effects on physiological functions and overall health. The beneficial commensals have several functions such as providing essential micronutrients, regulating the immune response, modulating enterocyte function, influencing metabolism, and preventing colonization by pathogenic microorganisms (5). The gut ecosystem is highly dependent on the human diet, as well as its composition, as the microbes metabolize and thrive on the consumed foods. Dietary fibers, microbiota accessible carbohydrates (MAC), and certain plant-based proteins are metabolized to short chain fatty acids (SCFAs). SCFAs exhibit anti-inflammatory properties, maintain mucosal integrity, and retain microbial diversity (6, 7). Imbalances in ratios of vital nutrients to dangerous toxins are implicated for several diseases, including cancer. Altered microbial diversity, impaired immune response, and release of carcinogenic or genotoxic substances are the major microbiome-induced mechanisms implicated for cancer pathogenesis (8). In this study, we aim to present emerging evidence on the dietary factors associated with the development of colorectal cancer (CRC). In addition, we also explored how healthy dietary modifications can restore functional colonic epithelium and prevent CRC.

## Gut Microbiome and Colorectal Cancer

**Figure 1:**
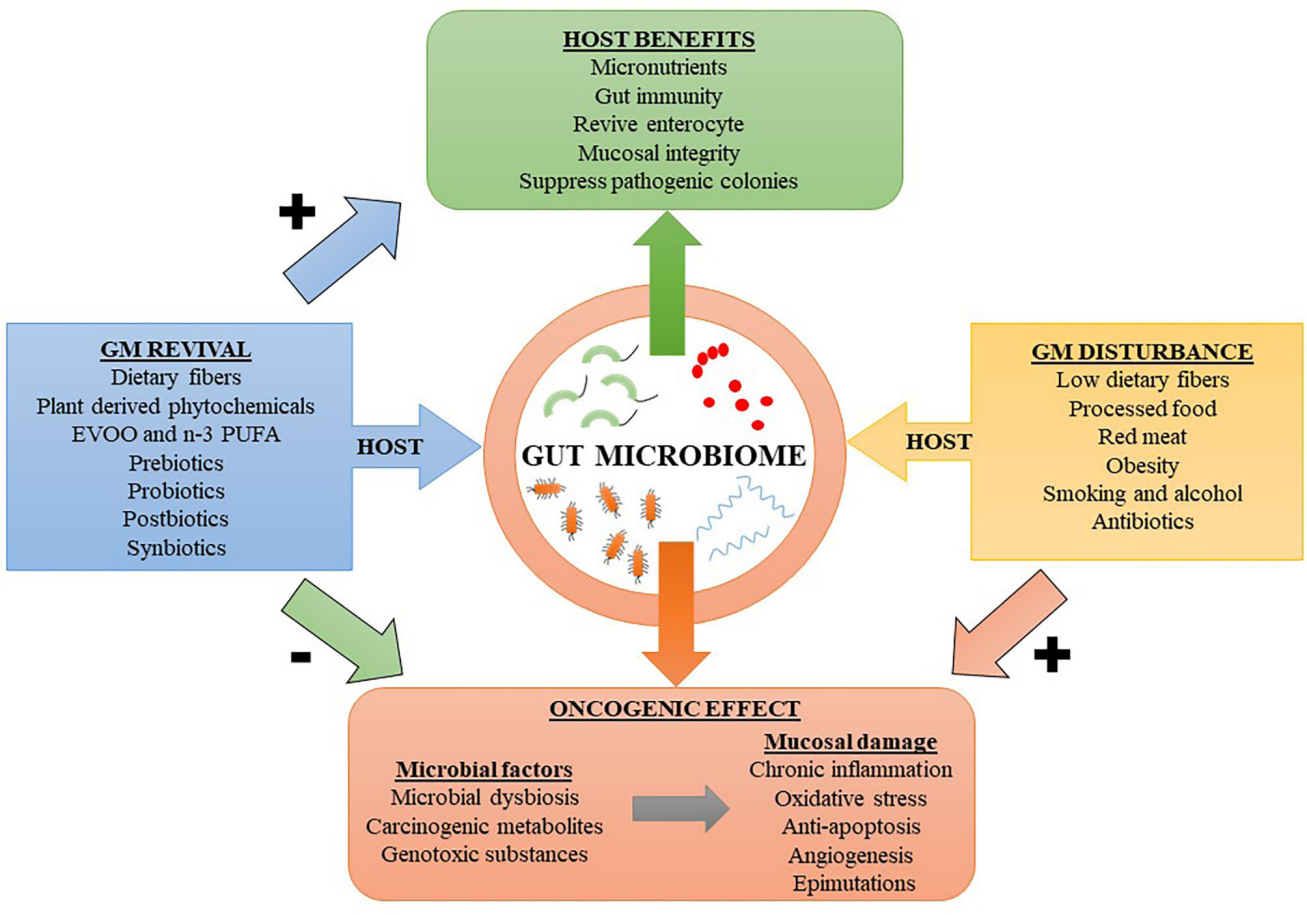
A host diet-outcome model showing the relation between host factors modulating the gut microbiome (GM). Dietary factors reviving the GM can result in beneficial effects to the host and prevent neoplastic changes in the colonic mucosa. While factors leading to GM disturbances can putatively increase the risk for colorectal cancer. EVOO, extra virgin olive oil; n-3 PUFA, omega-3 polyunsaturated fatty acid. Symbols: Positively associated (+); Negatively associated (–).

The gut microbiome can influence the development of CRC in several ways. Perturbations in the gut microbiota expose the GIT to inflammatory and genotoxic metabolites such as secondary bile salts, trimethylamine-N-oxide (TMAO), hydrogen sulfide (from sulfur containing amino acids), heme, nitrosamines, heterocyclic amines, and polyaromatic hydrocarbons, often resulting from consumption of red or processed meat and diet poor in fibers (9, 10). These dietary factors along with lifestyle factors such as smoking, alcohol and obesity increase the risk of oncogenic transformations in the colonic epithelial cells (1). Figure 1 shows a diet-outcome model that incorporates the host-microbe relationship and factors influencing their harmony. In the human body, most of the bacteria reside in the colon and an estimated 3.8 × 10^13^ bacteria could be found in a 70 kg “reference man” (11). In addition to natural gut defenses, human symbiotic microorganisms have additional roles in fighting pathogenic strains by stimulating the immune system. In turn, the immune system responds by producing a myriad of inflammatory mediators, chiefly consisting of anti-microbial peptides, inflammasomes, and cytokines [e.g., interleukin (IL)-22, IL-17, and IL-10] (12). Importantly, persistent activation of the immune system has its own adverse effects. Chronic inflammation can induce oxidative stress by producing reactive oxygen species (ROS), which have both cytotoxic and genotoxic effects, resulting in detrimental effects on intestinal mucosal cells (13). Inflammasomes produced by the innate immune system secondary to inflammation can lead to colitis and increase the risk for CRC (14). Moreover, inflammation-mediated persistent release of growth factors, suppression of apoptosis, and increased angiogenesis are additional factors which promote tumorigenesis (15). Carcinogenic metabolites or oncotoxins are produced due to alterations in microbial metabolism resulting from alerted dietary patterns such as consumption of processed and refined foods. These oncotoxins are implicated for promoting CRC (1). Yang et al. performed integrated metagenomic and metabolomic analysis and found that lower microbial diversity and increased production of cytotoxic polyamines such as cadaverine and putrescine are associated with increased risk for CRC (16). Diets higher in red and processed meats and lower in dietary fibers increases the predisposing factors for CRC (17). Indigestible dietary fibers reaching the lower GIT are metabolized by the gut microbiome into SCFAs such as acetate, propionate and butyrate, which has anti-inflammatory effect on the colonic mucosa (18). The mechanisms by which dysbiotic microbiome mediates CRC include increased microbial adherence to colon cells, downregulation of tumor suppressor genes, activation of oncogenes, induction of genotoxic effects on colonic enterocyte, and activation of angiogenesis (19). Thus, external factors can modulate the gut microbiome, resulting in either stimulatory or regulatory roles in priming the intestinal microenvironment toward or against tumorigenesis.

Poor microbial diversity is associated with increased risk for CRC (16, 20). Wu et al. reported abundance of *Helicobacter* spp. in right-sided and moderate to poorly differentiated CRCs, while *Firmicutes* phylum was higher in advanced CRCs with lymph node metastasis, compared to CRCs without lymph node metastasis (21). In colonic adenomatous polyposis (CAP), a precursor lesion to CRC, *Bacteroides* and *Citrobacter* taxa predominate, compared to *Weissella* and *Lactobacillus*, which are disproportionally low. The chief metabolites observed in the fecal samples of CAP patients were acetic acid and butyric acid, while healthy controls had higher levels of protective t10, c12-conjugated linoleic acid (not the same as dietary linoleic acid) (22). Conjugated linoleic acid such as c9, t11-CLA can be synthesized by natural gut colonizers such as probiotic Bifidobacteria species and strains of ruminal bacteria such as Megasphaera elsdenii produces t10, c12- conjugated linoleic acid (23, 24). Even though butyrate has pro-apoptotic and anti-proliferative role in CRC, it has paradoxically been shown to enhance polyp formation in *Apc*^*min*/+^*Msh2*^−/−^ (adenomatous polyposis coli^min/+^ and mutS homolog 2^−/−^) mice having defective mismatch repair (25, 26). Another bacterium that is highly implicated in CRC is *Streptococcus gallolyticus* subspecies *gallolyticus* (27). This bacterial strain carries a type VII expression system consisting of core genes expressing proteins which have potential pro-tumorigenic role in CRC through attachment to HT29 colon cancer cells and inducing subsequent proliferative changes. Deletion of the secretion system suppresses the protein expression related to bacterial attachment to the HT29 cells *in vitro* and decreased *Streptococcus gallolyticus* subspecies *gallolyticus* colonization in murine *in vivo* colon cancer models (27). This suggests that bacterial proteins produced by selective species can potentially exhibit pro-tumorigenic effects.

Certain pharmacological agents also modulate the colonic microbial diversity and alter the course of CRC. For example, Ternák et al. demonstrated that antibiotic therapy may have positive and negative correlation with development of several malignancies (28). In certain European regions, overconsumption of antibiotics such as penicillin and tetracyclines are associated with higher incidence of CRC, especially among females (28). Lee et al. reported that antibiotic therapy, either alone or in cocktail combinations, administered to murine colitis-associated cancer models decreased the bacterial load, suppressed inflammation, and impeded tumorigenesis in a drug-specific manner (29). This suggests that abnormal bacterial colonies can increase tumorigenesis and antibiotic therapies can plausibly modulate it. Anti-diabetic drug such as metformin produced significant changes in the gut microbiome of CRC patients with type 2 diabetes mellitus (T2DM). Comparative analysis among CRC patients with T2DM showed that metformin was associated with increase in *Firmicutes* and decrease in *Bacteroidetes* and *Fusobacteria* at the phyla level, while *Bifidobacterium* increased at genus level, whereas the abundance of pro-tumorigenic species *F. nucleatum* was decreased (30). Metformin treatment in T2DM has shown to increase the gut bacteria associated with enhanced production of SCFA such as butyrate and propionate which improve glucose metabolism and homeostasis (31). This suggest that metformin could improve gut microbiota that could have protective effects against CRC. Anti-diabetic drugs such as metformin induces changes in the gut microbiome of T2DM patients developing CRC. Comparative analysis showed that initiation of metformin in T2DM patients with CRC was associated with an increase in *Firmicutes* and decrease in *Bacteroidetes* and *Fusobacteria* at the phyla level while at genus level increase in *Bifidobacterium* with an associated decrease in *F. nucleatum* (30). Metformin treatment of T2DM has shown to enhance the microbial richness of the colon associated with enhanced production of SCFAs such as butyrate and propionate which potentially improves glucose metabolism and homeostasis (31). This suggest that initiation of metformin treatment in T2DM could be useful in recuperating altered gut microbiota, thereby imparting protective effects against CRC.

Five genera of microbes typically associated with CRC progression and reversed by metformin induction involves *Bacteroides, Streptococcus, Achromobacter, Alistipes, and Fusobacterium* (32). Passenger bacteria belonging to *Fusobacterium* genus, which reside in the oral cavity are associated with CRC progression (33, 34). *Fusobacterium nucleatum*, is mainly localized in the proximal colon and decreases in numbers from caecum to rectum, possibly due to anaerobic conditions. *F. nucleatum* is associated with more advanced and serrated forms of CRC (33–35). The abundance of *F. nucleatum* is affected by a number of environmental factors such as smoking, chronic periodontitis, and uncontrolled T2DM (36). In *Apc*^*min*^^/+^ CRC model mice, metformin suppressed the tumor growth induced by *F. nucleatum* colonization (32). Yu et al. reported that *F. nucleatum* directly targeted the TLR4-MYD88 (toll-like receptor 4-myeloid differentiation primary response 88) axis of the innate immune system to activate autophagy. Autophagic activity mediated by enhanced ULK1 (unc-51 like autophagy activating kinase 1) and ATG7 (autophagy related 7) expression improved cell survival and decreased chemotherapy-induced cytotoxicity (37). Though abundance of *F. nucleatum* in stool samples was positively associated with CRC, no significant associations were observed for adenomas (38). Activation of various pro-oncogenic pathways and pro-inflammatory mediators are possibly linked to the pathophysiology of *F. nucleatum* mediated CRC (35). FadA is an amyloid like virulence factor released by *F. nucleatum* in diseased states (39, 40). *In vitro* co-culturing HCT-116 and HT29 colon cancer cells with wild type (WT) *F. nucleatum* increased cell proliferation and DNA damage induced by fadA mediated activation of checkpoint kinase 2 (CHK2). Knockout of the *fadA* gene in *F. nucleatum* subsequently attenuated these proliferative changes induced in co-cultured HCT-116 and HT29 colon cancer cells (41). Furthermore, cancer tissue from *fadA*^−/−^ *F. nucleatum*-treated *Apc*^*min*^^/+^ mice had decreased expression of pro-proliferative CHK2. Similarly, recombinant fadA enhanced the proliferation of SW480 colorectal cancer cells *in vitro*, which was dose and time dependent (42). Among human CRC patients Kashani et al. observed that all CRC tissue biopsies were colonized with fadA positive *F. nucleatum*, indicating a strong relationship (43). This shows that fadA secreted by *F. nucleatum* is a key virulence factor and has a pro-tumorigenic role in CRC. Moreover, low dietary fiber intake putatively enhances the risk for *F*. *nucleatum* mediated CRC (35). Mehta et al. reported that healthy diet consisting of whole grains and dietary fibers are associated with decreased risk of *F. nucleatum* positive CRC (44). This suggest that decreasing *F. nucleatum* colonization in the lower GIT through healthy dietary changes could be a preventive measure against CRC. Wang et al. identified that *Eubacterium rectale* initiated chronic inflammation by activating downstream NF-κB signaling, which increased chemokine and cytokine production (45). Upregulation of NF-κB signaling pathway in CRC has shown to promote cancer growth by inducing cell proliferation, angiogenesis, inflammation, metastasis, and drug resistance (15). Collectively, these mechanisms demonstrate that inflammation induced by altered gut microbiota could trigger potential oncogenic pathways.

Certain pathogenic microbes residing in the proximal colon of CRC patients produces biofilm, which has pro-malignant potential (34). In familial adenomatous polyposis (FAP), which is a precursor lesion to CRC, colonization and invasion of the intestinal mucosa by carcinogenic toxin producing *Escherichia coli* and *Bacteroides fragilis* led to the formation of biofilms, which was associated with increase in the genes for bacterial toxins such as colibactin and enterotoxigenic *Bacteroides fragilis* (ETBF) toxin (46). Co-colonization of toxigenic *Escherichia coli* and *Bacteroides fragilis* into FAP model mice showed increased production of ETBF induced pro-inflammatory IL-17 and colibactin mediated DNA damage, which collectively accelerated the development of CRC (46). This shows that toxigenic bacterial species can enhance the risk of malignant transformation in benign colonic polyps. Therefore, evolving evidence show that altered gut microbiome could alter gut microbial crosstalk with the colonic mucosa and potentially enhanced the risk for CRC (Table 1).

**Table 1:** Plausible effect on the CRC tumorigenicity by relative abundance of diverse microbial taxa.

**Bacterium/taxa**	**Significance**	**Tumorigenicity in CRC (plausible)**	**References**
*Fusobacterium nucleatum*	↑ miR21 *via* TLR4/MyD88/NF-κB pathway	Pro-tumorigenic	(47)
	FadA mediated CHK2 promotes HCT-116 and HT29 colon cancer cell proliferation	Pro-tumorigenic	(41)
	Recombinant fadA induces proliferation of SW480 colorectal cancer cells	Pro-tumorigenic	(42)
	Metformin therapy in type 2 diabetics with CRC reduced the abundance of *F. nucleatum*	Pro-tumorigenic	(30)
	↑ Autophagy *via* ULK1 and ATG7	Pro-tumorigenic	(37)
	Pro-inflammatory foods associated with *F. nucleatum* positive CRC	Pro-tumorigenic	(48)
	NF-κB activation and synthesis of IL-1β, IL-6, TNF-α, and CXCL8	Pro-tumorigenic	(49)
	Increased unrestricted calorie intake during childhood	Pro-tumorigenic	(50)
	Associated with microsatellite instability (MSI) and CpG island methylator phenotype (CIMP)	Pro-tumorigenic	(51)
*Firmicutes* (phylum)	Cytotoxicity and genotoxicity inducing heme iron reduces its abundance	Anti-tumorigenic	(52)
*Proteobacteria* (phylum)	Increased abundance associated with food emulsifiers	Pro-tumorigenic	(53)
	Abundance enhanced by dietary heme iron	Pro-tumorigenic	(52)
*Helicobacter* spp.	↑ in moderate to poorly differentiated right sided colon cancer	Pro-tumorigenic	(21)
*Streptococcus gallolyticus* subspecies *gallolyticus* (strain)	Type VII expression system and ↑ bacterial attachment to HT29 colon cancer cells	Pro-tumorigenic	(27)
*Eubacterium rectale*	Chronic inflammation *via* NF-κB	Pro-tumorigenic	(45)
*Escherichia coli*	Colibactin induced DNA damage	Pro-tumorigenic	(46)
*Bacteroides fragilis*	ETBF mediated pro-inflammatory IL-17 production	Pro-tumorigenic	
*Bacteroides* (genus) *Clostridium* (genus)	Express bacterial sialidases which releases pro-inflammatory xenoantigen Neu5Gc from cell surface glycans thereby reducing chronic inflammation	Anti-tumorigenic	(54)
*Firmicutes, Bacteroidetes, and Proteobacteria* (phylum)	Express beta-glucuronidase and glycerol/diol dehydratase which metabolizes toxic heterocyclic amines from red meat	Anti-tumorigenic	(55)
*Ruminococcus bromii* *Bifidobacteriales (order)* *Turicibacteraceae* (family) *Lactobacillaceae* (family)	Shift to carbohydrate fermentation from protein metabolism when fed on both red meat and high amylose-resistant starch (in rat model)	Anti-tumorigenic	(56)
*Blautia* (genus)	Produces anti-inflammatory SCFA such as butyrate and propionate	Anti-tumorigenic	(57)
*Clostridium sporogenes, Clostridium subterminale* *Romboutsia lituseburensis*	*In vitro* produced SCFA on supplementation of antioxidants (ascorbic acid, glutathione and uric acids)	Anti-tumorigenic	(58)
*Bilophila wadsworthia* and *Erysipelotrichaeceae bacterium*,	Sulfur metabolizing bacteria associated with high microbial sulfur diet score and distal CRC	Pro-tumorigenic	(59)
*Faecalibacterium prausnitzii* ATCC 27768 strain *co-cultured with Bifidobacterium catenulatum* KCTC 3221 strain	Enhanced butyrate production and reduced IL-8 expression	Anti-tumorigenic	(60)
*Lactobacillus plantarum* *Lactobacillus reuteri* *Lactobacillus lactis*	Increase fecal butyrate levels associated with consumption of mango polyphenols	Anti-tumorigenic	(61)
↑*Lactobacillales* (order) ↓*Coriobacterales* (order)	Suppressed tumor size with curcumin intake *in vivo*	Anti-tumorigenic	(62)
*Lactobacillaceae* (Family) *Bifidobacteriaceae* (Family)	Probiotics	Anti-tumorigenic	(63)
*Clostridium XIVa*	Anti-inflammatory role		
*Akkermansia* (genus) *Desulfovibrio* (genus) *Anaerostipes* (genus)	Anti-inflammatory role	Anti-tumorigenic	(64)

*IL, interleukin; ATG7, autophagy related 7; CRC, colorectal cancer; CXCL8, C-X-C motif chemokine ligand 8; ETBF, enterotoxigenic Bacteroides fragilis; LMN, lymph node metastasis; NF-κB, nuclear factor kappa-light-chain-enhancer of activated B cells; SCFA, short chain fatty acid; TLR4, toll like receptor 4; TNF-α, tumor necrosis factor-α; ULK1, unc-51 like autophagy activating kinase 1; Symbol, ↑ (enhanced or increased)*.

## The Influence of Diet on Gut Microbiome and Colorectal Cancer Development

Dietary ingredients such as fibers, fat, and proteins fuel bacterial metabolisms which not only aid in the digestive process but also synthesize byproducts that have immense functional significance to the host. However, when this balance is impaired, toxic metabolites are generated by the gut microbes resulting in cytotoxic and genotoxic effects (Table 1). Moreover, diets rich in prebiotics and probiotics can increase the richness of microbiome by enhancing microbial diversity and nurturing the existing microbiota (65). Thus, the quality of diet delivered to the gut microbiota may be crucial for optimum health benefits. In the current era of processed food consumption, the gut biodiversity and chemical composition are profoundly affected, leading to chronic colonic inflammation, which increases the risk for CRC (9, 10, 66, 67). Processed meat consumption is associated with the risk of developing colorectal malignancies. Chemicals used for processing red meat react to form N-nitoso compounds (NOC), which are carcinogenic. This along with unhealthy dietary habits, obesity, heme iron, and associated alterations in gut microbiota enhance oncogenic changes in colonic mucosa (68). Viennois et al. reported that consumption of dietary emulsifiers such as carboxymethylcellulose (CMC) and polysorbate 80 (P80) alter the composition of the gut microbiome and increase the risk for intestinal inflammation and adenoma formation in *Apc*^*min*^ mice (69). Emulsifier consumption lowered the abundance of *Clostridia* (in both male and female mice) and increased the abundance of *Proteobacteria* (in male mice). *Proteobacteria* has been putatively linked to altered gut microbiome and risk for cancer (69, 70). In another study, Viennois et al. reported that CMC and polysorbate 80 induce low grade inflammation and alteration in the gut microbiota such as increase in *Bacteroidales* and decrease in *Clostridiales* (53). Long term consumption of emulsifiers increased fecal levels of bioactive products such as pro-inflammatory lipopolysaccharide and motility inducing flagellin. Consumption of food emulsifiers also increase the risk for developing metabolic syndrome and obesity, which are independent risk factors for CRC (71). This suggest that avoiding consumption of processed food could prevent colonization by harmful microorganisms, help in maintaining a healthy colonic mucosa, and decrease the risk for oncogenic changes.

Dietary factors such as higher levels of red meat, processed meat, refined sugar, alcohol, and high-fat diet, as well as lower levels of dietary fibers are implicated for mutagenic changes induced by the unhealthy microbiome and their metabolites (72, 73). Both red and processed meat are considered as potential risk factors for CRC because they alter the composition of gut microbiome (19). In a study done by Van Hecke et al. it was observed that nitrite-cured pork when cooked to very high temperatures increased the production of carcinogenic O^6^-carboxymethyl guanine DNA adduct when acted upon by fecal inoculums in an *in vitro* digestion model (74). In experimental rats, the heme iron from red meat decreased the number of operational taxonomical units (OTUs) in the colonic lumen, indicating altered gut microbiota. *Firmicutes* and *Deferribacteres* were specifically lowered, whereas *Bacteroidetes* and *Proteobacteria* counts were increased (52). Heme iron increases luminal lipid peroxidation, aldehydes, and ROS, leading to cytotoxic and genotoxic effects on colonic epithelium (52). Similarly, in a colitis model of mice fed with heme iron, Constante et al. reported depletion of *Firmicutes* phylum and overgrowth of the *Proteobacteria*. These mice had exacerbation of dextran sodium sulfate (DSS)-induced colitis and subsequently formed adenomas (75). However, clinical and epidemiological support for this outcome is limited. Based on a cohort study of over 49,000 women in Canada, there were no significant associations between iron, heme iron, or iron from meat and colorectal cancer (76). This evidence suggest that though red meat consumption could have detrimental effects on gut microbiota and colonic epithelium, epidemiological evidence is lacking, and further studies should explore this area.

Red meat is rich in sialic acid N-glycolylneuraminic acid (Neu5Gc), which following consumption gets incorporated into the cell surface glycans of endothelia and epithelia to behave as “xeno-autoantigens.” The human immune system produces anti-Neu5Gc antibodies against this antigen, triggering in chronic inflammation (77, 78). Neu5Gc is not expressed by human cells due to human-lineage specific genetic mutation in the enzyme cytidine monophospho-N-acetylneuraminic acid hydroxylase (CMAH). However, mammalian red meat such as sheep, goat and dairy cow are rich in Neu5Gc (78, 79). Circulating antibodies against Neu5Gc could initiate inflammation, which could potentially lead to cancers. For example, Samraj et al. reported that among *Cmah*^−/−^ deficient mice fed with Neu5Gc rich diet, there was higher levels of systemic inflammation and greater risk for hepatocellular carcinoma which was proportionate with levels of circulatory anti-Neu5Gc antibodies (80). Neu5Gc in diet increases the levels of *Clostridium* and *Bacteroides*, which efficiently express sialidases that release mucopolysaccharide from glycans (54). Although it is unclear whether this association is causational, microbes expressing Neu5Gc-specific sialidases can cleave Neu5Gc from red meat and prevent its incorporation into human tissue glycans (54). Such gut microbial species producing exo-sialidases may be protective for red meat consumers by potentially reducing Neu5Gc triggered inflammation. In addition, *Firmicutes, Bacteroidetes*, and *Proteobacteria* phyla can produces enzymes such as beta-glucuronidase and glycerol/diol dehydratase, which can metabolize heterocyclic amines from red meat into less toxic products, thus proving protective against CRC (55). These findings suggest that bacterial enzymes present in the microbiome could potentially metabolize pro-inflammatory substances and harmful toxins to lesser toxic metabolites, thereby decreasing the risk for inflammation and carcinogenesis.

Though evidence show that red meat may contribute to carcinogenesis *via* microbial alterations, the associations are weak. A review of 35 prospective studies showed that the association between red meat and colorectal cancer was minimal, and the highest relative risk was below 1.50 and not statistically significant (81). An alternate explanation is that specific combinations of foods may alter the beneficial vs. harmful effects associated with colonic microbiome. For example, when rats were concomitantly fed both red meat and high amylose-resistant starch, there was a shift in the gut metabolism from predominantly protein fermentation to a combination of both protein and carbohydrate fermentation. This was associated with increase in *Ruminococcus bromii, Bifidobacteriales, Turicibacteraceae*, and *Lactobacillaceae* in the gut microbiome (56). This change in taxonomical traits of the gut microbiome was associated with decreased expression of pro-oncogenic miR17-92, which imparted protective effects against CRC (56). Similarly, processed meats that were fortified with a prebiotic polysaccharide inulin, increased the abundance of *Blautia* genus, which increased the production of protective SCFAs such as propionate and butyrate (57). This was associated with decrease in colonic polyps in experimental rats. In addition, shifting to a fish-inclusive vegetarian diet (or pesco-vegetarian diet) might have potential benefit over a standard western diet due to favorable changes in gut microbiome taxonomical traits (82). Similarly, Orlich et al. reported that among all forms of mixed vegetarian and non-vegetarian diets, pesco-vegetarian diet holds the least risk for developing CRC (83). Collectively, it can be summarized that consumption of specific combination of foods could decrease the level of toxicity on the colonic epithelium and correspondingly decrease the risk for development of CRC.

Dietary constituents significantly modulate chronic inflammation by regulating the immune response. In a retrospective study, Liu et al. reported that CRC patients who consumed inflammatory foods had higher abundance of *F. nucleatum* in their cancer biopsies (48). The inflammatory potential of 18 food items were calculated based on the empirical dietary inflammatory pattern (EDIP) scoring system, which relies on the plasma levels of IL-6, C-reactive protein (CRP), and TNF receptor superfamily member 1B (TNFRSF1B) (84). Higher scores indicate inflammatory, while lower scores indicate anti-inflammatory effects. Foods with higher EDIP such as refined grains, red and processed meats, and carbonated beverages were associated with *F. nucleatum* positive CRC (48). Conversely, consumption of anti-inflammatory foods such as whole grains and fiber rich diets were associated with lower risk of developing *F. nucleatum* positive CRC (44). Fermented foods such as yogurts are protective to the colonic mucosa and maintain microbial diversity, which decreases the risk for CRC, especially in the proximal colon (85). Yogurts supplemented with lyophilized jabuticaba (*Myrciaria jaboticaba*) seed extract have strong prebiotic, antioxidant, and anti-cancer effects. These supplements, when fed to CRC rat models improved gut microbiome quality and increased the immune response and cytotoxic effects on colon cancer cells (86). This suggests that yogurt and other probiotics could be healthy supplements for the gut and its microbial ecosystem.

Antioxidant consumption is important for the survival of certain bacterial strains in the GIT. Decreased levels of antioxidants such as ascorbic acid, glutathione, and uric acids could be lethal for anaerobic gut bacterial species such as *Clostridium sporogenes, Clostridium subterminale*, and *Romboutsia lituseburensis*. Supplementation of these antioxidants in controlled aerobic condition *in vitro* resulted in production of protective SCFAs such as propanoic, butanoic, isobutanoic, and isopentanoic acids (58). This shows that supplementation of antioxidants decreases oxidative stress and enhances survival of anerobic microbes which produce SFCAs. SCFAs such as butyrate produced by the anaerobic species have protective effects in CRC (87). Among CRC survivors, consumption of legumes such as navy beans increased the production of beneficial metabolites such as such as piperidine, N-methylpipecolate, vanillate, and 2-aminoadipate. Gut microbes metabolized the indigestible substrates present in cooked navy beans and produced a total of 237 beneficial metabolites (88). In addition, navy bean consumers had 5.25 times higher levels of ophthalmic acid, which is associated with glutathione metabolism. Ophthalmic acid has an important role in detoxifying xenobiotics such as carcinogens and decreasing oxidative stress, thereby imparting protective effects against cancer (89–91). Individuals with diets deficient in dietary fibers and high in processed meat and sugary beverages show abundant colonization by sulfur-digesting bacteria such as *Bilophila wadsworthia* and *Erysipelotrichaeceae*. This is associated with an increase in risk for distal colon and rectal malignancies (59).

*Glycyrrhiza uralensis* polysaccharide (GCP) extracted from licorice impedes tumor growth and metastasis in mice inoculated with murine colon cancer (CT-26) cells. This is achieved by modifying the composition of gut microbiome such as increased levels of *Enterorhabdus, Odoribacter, Ruminococcaceae_UCG_014, Ruminococcaceae_UCG_010, Enterococcus*, and *Ruminiclostridium_5* (92). Similarly, polysaccharides extracted from jujube was associated with decreased inflammation in mouse colon cancer models, most likely due to an associated decrease in *Firmicutes* and *Bacteroidetes* taxa in the gut microbiota (93). Similarly, combinations of *Ganoderma lucidum* polysaccharides and *Gynostemma pentaphyllum* saponins decreased colonic inflammatory and precancerous changes in *Apc*^*min*^^/+^ mice. Together, they enhanced microbial richness by increasing SCFA producing microbes and decreasing sulfur digesting microbes (94). This suggests that certain plant and fungi-based products may be effective prebiotics and exert protective effects on the colonic epithelium.

Alcohol consumption is associated with alteration in gut microbiota that potentially accelerates CRC carcinogenesis. Alcohol is metabolized by the gut microbiota into toxic intermediates leading to colonic carcinogenesis *via* DNA-adducts, oxidative stress, epimutations, loss of epithelial barrier functions, and immunomodulations (95). These effects can be potentiated and aggravated by poor nutrition and chronic smoking status; covariates commonly associated with alcohol consumption. The microbiota in alcoholics have decreased levels of beneficial organisms such as *Bacteroides* and *Ruminococcus* and increased levels of harmful organisms such as *Streptococcus* (96). Integrated analysis using 16S rRNA (ribosomal RNA) gene analysis data and epidemiological characteristics by Kim et al. showed that alcohol consumption increased abundance of *F. nucleatum* in the gut which is associated with increased risk for CRC (97). Among alcoholics, deficiency of beneficial obligate anaerobe OTUs was demonstrated through decreased production of acetaldehyde in stool samples that were treated with specific quantities of ethanol under experimental conditions. This implies that restriction of alcohol consumption could potentially prevent genotoxic insults on colonic mucosa.

## The Effects of Dietary Interventions on Colorectal Cancer

**Figure 2:**
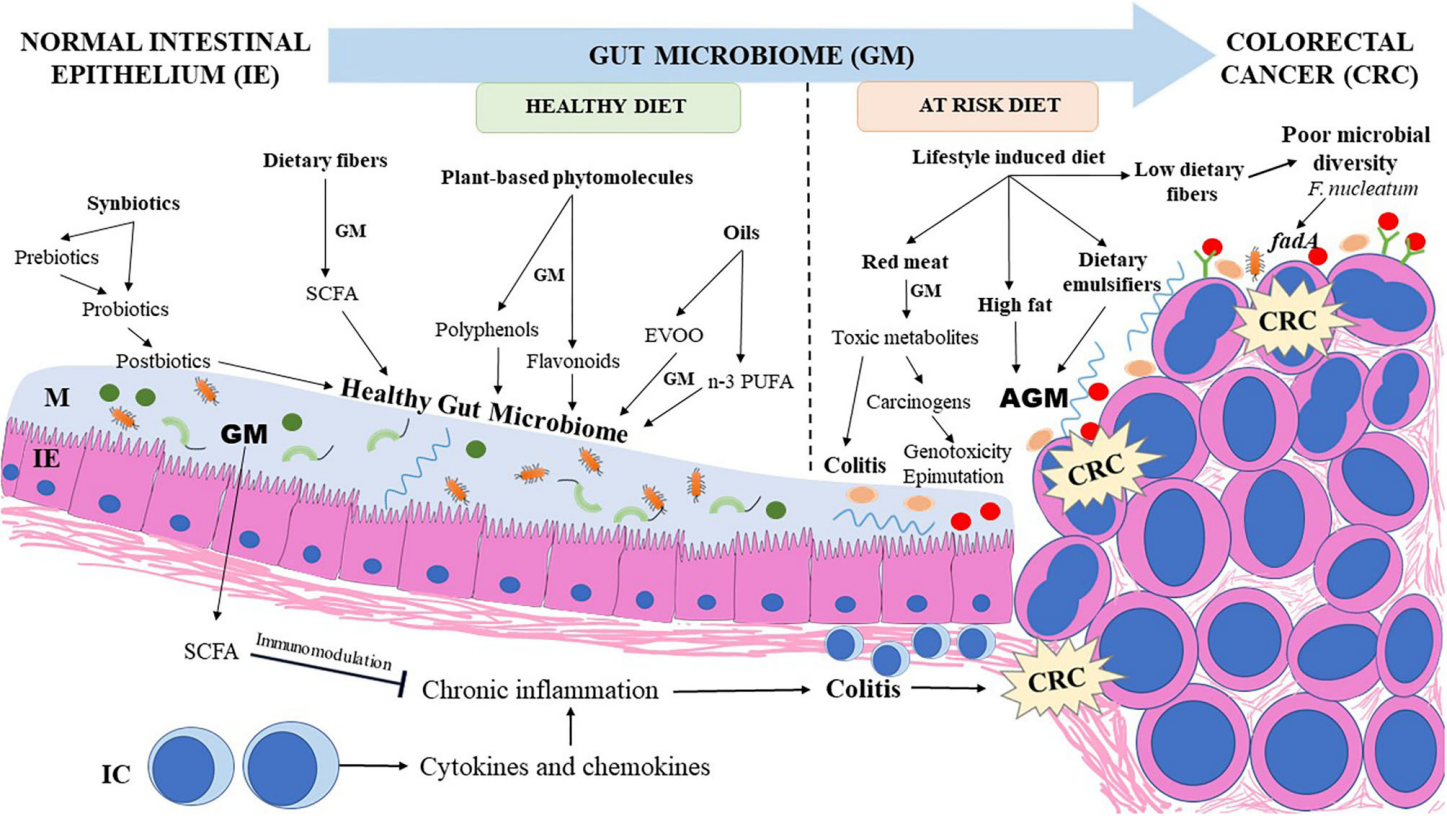
Influence of dietary factors on gut microbiome and its impact on CRC development. Dietary fibers, plant based phytomolecules, synbiotics, postbiotics, and essential oils such as n-3 PUFA (from marine sources) and EVOO chiefly support a healthy microbiome richness which provides useful metabolites protective to the colonic mucosa. Lifestyle induced changes in dietary pattern enhance the risk of DYB and aggravate colonic inflammation which progresses to CRC. CRC, colorectal cancer; AGM, altered gut microbiome; EVOO, extra virgin olive oil; GM, gut microbiome; IC, immune cells; IE, intestinal epithelium; M, mucus; n-3 PUFA, omega-3 polyunsaturated fatty acids; SCFA, short chain fatty acids.

Dietary fibers provided by plant-based diets are not digested by the human intestinal enzymes and reach the colon unchanged. Colonic bacteria express enzymes that metabolize and ferment soluble dietary fibers into useful metabolites such as SCFAs which play a major role in decreasing colonic mucosal inflammation and lowering the risk for CRC (98, 99). Butyrate has an inhibitory effect over the histone deacetylases (HDAC) enzymes, resulting in enhanced expression of genes which arrest the cell cycle (100). Butyrate also serves as an energy source for normal enterocytes; however, rapidly dividing CRC cells are dependent on glycolysis rather than butyrate utilization for energy needs (101). Co-culturing certain bacterial strains in animal models enhanced production of butyrate and improved the SCFA-mediated protection against CRC. *Faecalibacterium prausnitzii* ATCC 27768 strain co-cultured with *Bifidobacterium catenulatum* KCTC 3221 and supplemented with fructooligosaccharides in anaerobic conditions significantly enhanced butyrate production (60). Exposing the co-culture supernatant to HT29 colon cancer cells and RAW 264.7 macrophages decreased the release of pro-inflammatory cytokine IL-8 *in vitro* (60). The supernatant from the co-cultured bacteria also enhanced acetate, propionate, and butyrate levels in the caecum of the DSS-induced colitis in mice model as well as decreased gene expression of IL-8, suggesting the anti-inflammatory effect of these bacterial species *in vivo* (60). Butyrate was also shown to increase the extracellular tight junction protein complexes in an *Apc*^*min*^^/+^ mice model (102). This highlights the potential role of butyrate in decreasing the risk for CRC. Figure 2 illustrates the effects of dietary factors on the gut microbiome and their impact on CRC development.

Diet-derived phytochemicals, such as polyphenols and flavonoids, have protective effects on the colonic mucosa (103, 104). Most of the ingested polyphenols present in plant-based diets and their derivatives reach the colon unaltered and are metabolized by intestinal bacteria to more active substances which decrease oxidative stress, inflammation, and tumorigenesis (103). Polyphenols also act on the gut microbiota to enhance the proliferation of beneficial strains and inhibit pathogenic strains. Polyphenols increase the growth of probiotics and butyrate-producing microbiota such as *Lactobacillus* and *Bifidobacterium*, which inhibit inflammation, alleviates colitis, and decrease the risk for CRC (105). Polyphenols such as epigallocatechin-3-O-gallate and theaflavins present in tea extracts exert anti-inflammatory effects on *F. nucleatum*-induced inflammatory bowel disorders, thereby decreasing the risk for CRC (49). These anti-inflammatory effects are mediated by inactivation of NF-κB, as observed in U937-3xκB-LUC cell line (U937 cell line transfected with luciferase gene and coupled to three NF-κB binding sites). This results in decreased production of pro-inflammatory cytokines such as IL-1β, IL-6, tumor necrosis factor-α (TNF-α), and C-X-C motif chemokine ligand 8 (CXCL8). Polyphenols present in berries function as prebiotics and improve microbial richness in the form of *Bifidobacterium, Lactobacillus* and *Akkermansia*. Berry polyphenols also modulate the production of cytokines which alleviate inflammation and decrease the viability and proliferation of CRC cells (106). Polyphenols present in mango pulp such as gallotannins and gallic acid exhibit anti-inflammatory effects on the intestinal mucosa. In humans, consumption of mango pulp decreases pro-inflammatory cytokines such as IL-8, growth-regulated oncogene (GRO), and granulocyte macrophage colony-stimulating factor (GM-CSF). Mango polyphenols increase the abundance of *Lactobacillus plantarum, Lactobacillus reuteri*, and *Lactobacillus lactis*, as well as increase butyrate levels in feces (61). Date palms, another source of polyphenols and fibers, do not significantly alter gut microbiota or raise SCFAs in healthy volunteers, but decrease genotoxicity, fecal ammonia levels, and aid in bowel movements, thereby decreasing the risk for CRC (107). Similarly, administration of polyphenol-rich green tea extracts to human volunteers enhanced *Firmicutes* to *Bacteroidetes* ratio and SCFAs producing gut microbes (108). Thus, polyphenols are natural plant products which may have a potent role in reshaping the gut microbiome and thereby decrease the risk for CRC.

Curcumin, a natural product from *Curcuma longa* plant, is a polyphenol with a significant role in decreasing inflammation, oxidative stress, and alterations in gut microbiome (109). Similar to other polyphenols, curcumin is also subjected to bacterial metabolism, resulting in production of useful metabolites which have protective effects in CRC and also has key role in reviving beneficial microbial strains in the gut (110). IL-10-deficient CRC mice models on a curcumin-based diet demonstrated improved taxonomic profiles of gut microbiota, such as an abundance of *Lactobacillales* and lower levels of *Coriobacterales*. This was also associated with a reduction in tumor size and complete elimination of macroscopic lesions. In addition, there was restoration of β-catenin on plasma membranes, but the effect on mucosal inflammatory responses was limited (62). Farhana et al. reported that a combination of essential turmeric oil-curcumin and tocotrienol-rich fraction of vitamin E isomers effectively reduced the proliferation of colon cells (HCT-116 and HT-29 cells) *in vitro* and suppressed the growth of mice xenograft formed of HCT-116 cells *in vivo* (63). Bacterial 16S rRNA gene profiling revealed that turmeric oil-curcumin and tocotrienol treatment resulted in a significant increase in *Lactobacillaceae* and *Bifidobacteriaceae*, along with elevated *Clostridium* cluster XIVa, which produced an anti-inflammatory environment. In addition, the abundance of phylum *Bacteroidetes* and *Firmicutes* were relatively decreased. Collectively, this evidence supports the potential role of curcumin in combination with other natural substances in decreasing the risk for CRC.

Flavonoids are polyphenols abundant in fruits and vegetables and impart their natural colors (111). The gut microbiota and associated enzymes convert the flavonoids into bioactive metabolites, resulting in anti-inflammatory, antioxidant, and anti-tumor effects (111). Neohesperidin, a flavonoid which is abundant in citrus fruits, imparts tumoricidal activity in *Apc*^*min*/+^ CRC mice models by inhibiting angiogenesis and promoting apoptosis (112). Fecal microbiota transplantation from neohesperidin treated mice decreased colonic tumorigenesis *in vivo*, mainly by modulating the gut microbiota composition. Neohesperidin treatment increased the abundance of phylum *Firmicutes* and *Proteobacteria*, while decreased *Bacteroidetes* (112). Black raspberry anthocyanins are another group of protective flavonoids that decreased tumorigenesis in colitis-associated CRC model mice by inducing epigenetic changes (50). Pan et al. reported that consumption of raspberry anthocyanin increased the abundance of anti-inflammatory bacterial genus such as *Akkermansia* and *Desulfovibrio* as well as butyrate producing *Anaerostipes*. However, alteration in the microbial composition was achieved only on consumption of whole raspberries (64). Thus, consumption of flavonoids abundant in plant-based diets improved microbial richness and decreased CRC growth.

Olive oil, an essential component of the Mediterranean diet, is high in monounsaturated fatty acids, squalene, phytosterols, and phenols (113). Phenolic derivatives of some of these nutrients are further metabolized by the gut microbiota into chemopreventive active substances. Consumption of extra virgin olive oil (EVOO) has beneficial effects on the mucosal cells, compared to sunflower and coconut oil. In an experimental mice model study, high-fat diet with sunflower and coconut oil produced perturbations in gut microbiota and inflammatory changes (73). EVOO also decreased harmful microbes from the genus *Enterococcus, Staphylococcus, Neisseria*, and *Pseudomonas* (73). This suggests that diets rich in EVOO could be protective against CRC, compared to other oils. N-3 polyunsaturated fatty acid (PUFA) in combination with fermentable dietary fibers have protective role in pathways related to programmed cell death and epigenetic dysregulation observed in CRC (114). In a randomized control trial, consumption of n-3 PUFA increased butyrate-producing bacteria such as *Bifidobacterium, Roseburia*, and *Lactobacillus*, suggesting that it had a role in reducing inflammation and CRC risk (115). However, it is noteworthy that PUFA and sphingolipids were altered in the fecal metabolomic profile of patients with adenoma-carcinoma and resulted in a preponderance of harmful species such as *Firmicutes* and *Bacteroidetes* (116). Moreover, Kraja et al. reported that n-3 PUFA from non-marine sources and reduced dietary fiber consumption increased the risk for CRC (117). It can be postulated that the possible benefits of n-3 PUFA on colonic mucosa are effective only when it is obtained from marine sources and consumed in combination with dietary fibers. Collectively, it suggests that careful selection of lipids in diet, especially EVOO and n-3 PUFA, is necessary for optimizing healthy colonic mucosa.

The combination of prebiotics and probiotics is known as synbiotics. Consumption of synbiotics is considered an active intervention to improve the quality of gut microbiome for preventing CRC. Synbiotics work by enriching the gut microbiome and the microbial strains which impart protective mucosal functions such as decreasing inflammation, preventing uncontrolled proliferation, preventing altered immune responses, lowering production of toxic metabolites, and lowering oxidative stress (118). In an experimental *in vitro* chip-based model (HuMiX gut-on-a-chip), synbiotics downregulated oncogenic signaling pathways (in Caco-2 cells). The synbiotics consists of probiotic *Lactobacillus rhamnosus* Gorbach-Goldin strain and a complex prebiotic formula simulating high fiber diet which was prepared from non-digestible carbohydrates and other prebiotics such as arabinogalactan, xylan and soy (119). Synbiotic administration not only decreased the production of oncometabolite lactate and suppressed drug resistance genes in colon cancer-derived cells, but also increased SCFAs such as acetate and formate (119). A new synbiotic combination of *Lactobacillus gasseri* 505 and *Cudrania tricuspidata* leaf extract in fermented milk decreased *Staphylococcus* and increased *Lactobacillus, Bifidobacterium*, and *Akkermansia*, thus increasing protective effects in DSS/azoxymethane (AOM) induced colitis-CRC model mice. This *in vivo* intervention decreased tumor proliferation and inflammation (marked by decreased levels of TNF-α, interferon (IFN)-γ, IL-1β, IL-6, inducible nitric oxide synthase, and cyclooxygenase-2) and led to upregulation of anti-inflammatory cytokines IL-4 and IL-10 (120). Praveen et al. developed raindrop candy consisting of polysaccharides extracted from Indian seaweed (*S. wightii, E. compressa*, and *A. spicifera*) and probiotic species *L. plantarum* NCIM 2083. These seaweed polysaccharides demonstrated anti-cancer effects on RAW 264.7 macrophage and HT-29 human colon cancer cell line *in vitro* (121). Thus, synbiotics could be novel therapeutic approaches to strengthen the gut microbiome for protective effects against CRC through alleviating inflammation and preventing tumorigenesis. However, due to the concerns of consuming live microbial species and uncertainties about the net effects of its colonization in the gut, postbiotics are gaining increasing attention as a paradigm shift (122, 123). Postbiotics are composed of inactivated microbial cells or their components with or without microbial metabolites, which shows beneficial effects on human health (124). Lactic acid bacteria (LAB) which are found in fermented foods are an essential source of postbiotic metabolites (PM) with physiological effects and health benefits including opposing effects on tumorigenesis (125). The cancer opposing effects of postbiotics are achieved through improving the gut microbiota, reprogramming the immune function, enhancing response to CRC treatment, and increasing antioxidant, anti-proliferative, and anti-inflammatory actions (126). Postbiotic metabolites (PMs) produced from *Lactobacillus plantarum* isolated from Malaysian fermented food, *Tapai Ubihas*, has remarkable anti-cancer effects such as enhanced cytotoxicity and anti-proliferative action (127, 128). PMs from six different strains of *L. plantarum* were evaluated for their anti-cancer effect on cells lines of five different cancers, which included breast, colorectal, cervical, liver, and leukemia. PMs obtained from two strains RG14 and I-UL4 effectively inhibited proliferation of HT29 colon cancer cells. Some PMs show anti-proliferative effects when added in specific microenvironments. For example, PM from the cell free supernatant of *Streptococcus salivarius* M18 cultured on prebiotic inulin displayed potent inhibitory effect on colon cancer cell proliferation through increasing extracellular acidity within the tumor (129). Postbiotics like SCFA and tryptophan have shown to downregulate both the adaptive and humoral immunity in inflammatory conditions such as inflammatory bowel diseases (130). This shows that postbiotics can not only prevent cancer proliferation, but also ameliorate mucosal inflammation. Thus, consumption of dietary fibers and diet-derived factors such as phytochemicals and essential fatty acids, as well as prebiotics, probiotics, and postbiotics could be considered a multifactorial protective approach against CRC. Table 2 presents the studies on dietary factors influencing the gut microbiome and its effect on the colonic mucosa and CRC progression.

**Table 2:** Studies showing the effect of dietary factors influencing the gut microbiome and its impact on the colonic mucosa and CRC progression.

**References**	**Human/*in vivo*/*in vitro***	**Dietary factors or intervention**	**Influence on gut microbiome/bacterial species or strain**	**Impact on colon/CRC**
Constante et al. (75)	*In vivo* (mice)	Heme iron (red meat)	↓*Firmicutes* ↑ *Proteobacteria*	↑ DSS induced Colitis ↑ Colitis induced adenoma
Fernández et al. (57)	*In vivo* (rat)	Processed meat mixed with polysaccharide inulin (functional food)	*↑ Blautia*	CRC prevention ↑ SCFA production ↑ Anti-inflammatory action
Lagha and Grenier (49)	*In vitro* (U937-3xκB-LUC cell line and PMA treated U937 human monocytes)	Epigallocatechin-3-O-gallate and Theaflavins (Tea polyphenols)	↓*Fusobacterium nucleatum*	↓ Inflammation ↓ NF-κB activation
Kim et al. (61)	Human	Mango pulp polyphenols	↑*Lactobacillus*	↓ Intestinal inflammation ↓ IL-8, GRO and GM-CSF
Gong et al. (112)	*In vivo* (mice)	Neohesperidin (Flavonoid)	↑*Firmicutes* ↑*Proteobacteria* ↓ *Bacteroidetes*	↑ Apoptosis ↓ Angiogenesis
Chen et al. (50)	*In vivo* (mice)	Black raspberry anthocyanin (Flavonoid)	↑*Eubacterium rectale* ↑*Faecalibacterium prausnitzii* ↑ *Lactobacillus*	↓ Tumorigenesis ↓*SFRP2* promoter methylation
Pan et al. (64)	*In vivo* (rat)	Black raspberry anthocyanin (Flavonoid)	*↑ Akkermansia* *↑ Anaerostipes* *↑ Desulfovibrio*	CRC prevention
Rodríguez-García et al. (73)	*In vivo* (mice)	Extra virgin olive oil	↑ Firmicutes:Bacteroidetes ↑*Akkermansia* ↓*Enterococcus* ↓*Staphylococcus* ↓*Neisseria* ↓ *Pseudomonas*	↓ Alteration in gut microbiota ↑ Anti-inflammatory effect
Watson et al. (115)	Human	n-3 PUFA	↑*Bifidobacterium* *↑ Roseburia* *↑ Lactobacillus*	CRC prevention (Increase butyrate producers)
Kim et al. (60)	*In vitro* (co-culture)	Fructooligosaccharides	*Faecalibacterium prausnitzii* ATCC 27768 strain and *Bifidobacterium catenulatum* KCTC 3221 strain	↑ Butyrate production
	*In vivo* (mice)	Fed with *F. prausnitzii* and *B. catenulatum* co-culture supernatant	↑*Akkermensia* ↑ Verrucomicrobiales	↑ Acetate, propionate and butyrate in the caecum ↑*Il8* gene expression
Yuan et al. (108)	Human	Green tea extracts (Polyphenols)	↑ Firmicutes:Bacteroidetes ↑ SCFA producers ↓ *Fusobacterium*	CRC prevention
Farhana et al. (63)	*In vivo* (mice)	Essential turmeric oil-curcumin and vitamin E isomers	↑*Lactobacillaceae* *↑ Bifidobacteriaceae* *↑ Clostridium XIVa*	↓ CRC proliferation ↑ Probiotic action ↑ Anti-inflammatory effect
Greenhalgh et al. (119)	*In vitro* (HuMiX gut-on-a-chip model with synbionts)	Simulated high fiber diet (Prebiotic)	*Lactobacillus rhamnosus* Gorbach-Goldin (Probiotic)	CRC prevention ↓ Oncogenic pathways ↓ Lactate production ↓ Chemoresistance
Mehta et al. (44)	Human	Prudent diet (Whole grain and dietary fiber)	↓*Fusobacterium nucleatum*	↓ CRC risk
Oh et al. (120)	*In vivo* (mice)	*Cudrania tricuspidate* extracts in fermented milk (Prebiotic)	*Lactobacillus gasseri* 505 (Probiotic)	*↑ Lactobacillus* ↑*Bifidobacterium* ↑*Akkermansia* ↓ Inflammatory cytokines ↑ Anti-inflammatory cytokines
Li et al. (51)	Human	Childhood calorie restriction	↓*Fusobacterium nucleatum*	↓ CIMP and ↓MSI which influences prognosis of CRC
Sobhani et al. (131)	*In vivo* (mice)	Fecal microbiota transplantation (of CRC subjects to germ free mice)	↓ Coprococcus ↑ Bacteroides	Enhanced DNA mutation and hypomethylation involving genes of pro-oncogenic Wnt and Notch pathway in mice
Alrafas et al. (132)	*In vivo* (mice)	Resveratrol (plant stilbenoid)	↑ SCFA (butyrate and iso-butyrate) producers	CRC prevention ↓ HDAC ↑ Foxp3 ↑ Treg cells and IL-10 ↓ Th1 and Th17 cells

*CIMP, CpG island methylator phenotype; CRC, colorectal cancer; DSS, dextran sulfate sodium; Foxp3, forkhead box P3; GM-CSF, granulocyte-macrophage colony-stimulating factor; GRO, growth-regulated oncogene; HDAC, histone deacetylase; IL-8, interleukin-8; MSI, microsatellite instability; NF-κB, nuclear factor kappa-light-chain-enhancer of activated B cells; PUFA, polyunsaturated fatty acids; SCFA, short chain fatty acid; SFRP2, secreted frizzled related protein 2; Symbols, Enhanced (↑); Reduced (↓)*.

## The Diet-Gut Microbiome-Epigenetics Axis

**Figure 3:**
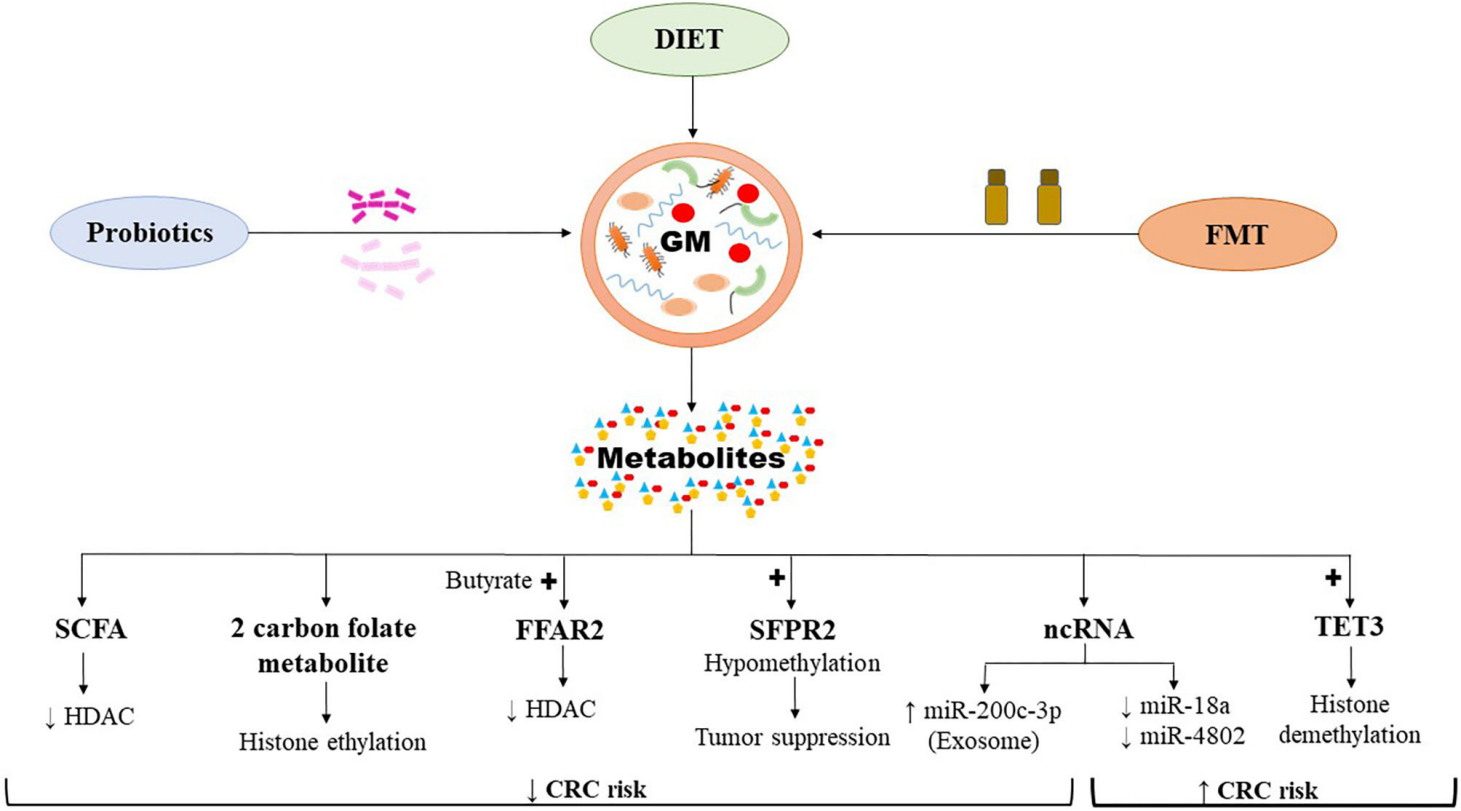
The host-gut microbiome influencing the CRC associated epigenetics. The resulting gut microbial metabolites can induce pro-oncogenic or onco-suppressive effects on CRC by modulating epigenomics. CRC, colorectal cancer; FFAR2, free fatty acid receptor 2; FMT, fecal microbiota transplantation; GM, gut microbiome; ncRNA, non-coding RNA; SCFA, short chain fatty acids; SFPR2, secreted frizzled related protein 2; TET3, ten eleven translocation 3. Symbols: Enhanced (↑); Reduced (↓); Activation (+).

Cancer is triggered by a multitude of factors that destabilize the genetic regulatory mechanisms controlling the cell proliferation events. Apart from mutations occurring in the tumor suppressor genes, or protooncogenes leading to either loss or gain of resulting protein function, epigenetic changes also transform the transcriptomic profile and the genomic landscape resulting in oncogenic CRC traits (Figure 3). Epigenetic dysregulation, otherwise known as epimutations, commonly occur by promoter methylation/demethylation of CpG islands, histone acetylation/deacetylation, or by non-coding RNA such as miRNA which alter the expression of genes involved in cellular growth, differentiation, and metabolism (133). The gut microbiome is unique in the sense that it carries millions of genes which execute functions exotic to the human genome and their metabolic activities depend on the substrate presented to them by the host diet, thus establishing a symbiotic relationship. However, this symbiosis comes at a cost, as impaired nutrition can result in the synthesis of harmful metabolites which potentiates the susceptibility of the host genomic architecture to genotoxicity (133).

The SCFAs, which are bacterial metabolites produced by digestion of dietary fibers by gut microbes, lower certain epigenetic alterations in enterocytes associated with CRC (98, 99). SCFAs, such as butyrate, protect the genetic and epigenetic architecture of enterocytes through multiple mechanisms (98, 134). The foremost includes its anti-inflammatory action, whereby it alleviates colonic mucosal inflammation and directly decreases the risk for CRC. Butyrate upregulates the activity of T-regulatory (T-reg) cells which exert an inhibitory effect on pro-inflammatory cytokine production and thereby blocking pro-oncogenic pathways (135). Butyrate has an inhibitory effect over the HDAC enzymes, which results in enhanced expression of genes arresting the cell cycle (100). Free fatty acid receptor 2 (FFAR2), activated by SCFAs such as butyrate, is known to suppress inflammation and prevent epigenetic dysregulation in CRC (136). Loss of FFAR2 in a DSS/AOM treated *Apc*^*min*^^/+^ colitis-CRC model mice led to overexpression of HDAC, which is mediated through an upregulation of CREB (cAMP-response element binding protein) (136). This resulted in an epigenetic downregulation of immunomodulating genes such as *Sfrp1*, Dickkopf-related protein 3 (*Dkk3*), and suppression of cytokine signaling 1 (*Socs1*), which were collectively associated with enhanced infiltration of the colonic mucosa and tumor tissue by the neutrophils. The study demonstrated that the epigenetic dysregulation induced by loss of *Ffar2* resulted in enhanced colonic inflammation, progressing into adenoma and adenocarcinoma formation (136). One carbon metabolism mediated by S-adenosyl methionine (SAM) transfers a methyl group to the CpG islands in the DNA promoter region which affects the gene expression and is of significance in CRC (137). Dietary consumption of methyl donors such as methionine, betaine, and choline as well as B vitamins (folate, B2, B6, and B12) have protective effects on the composition of gut microbiota (138). Deficiency of these nutrients decreases the abundance of beneficial organisms such as *Akkermansia, Roseburia*, and *Faecalibacterium*. Thus, the host genome is exposed to a number of dietary and microbial metabolites, which in turn would determine the beneficial vs. harmful effects on the pathogenesis of CRC.

The absence of caloric restriction during childhood may negatively impact microbial composition and may contribute to epigenetic dysregulation and development of CRC later in adulthood (51). Subjects who were energy restricted during their childhood had decreased abundance of pathogenic species such as *F. nucleatum, Bacteroides fragilis*, and *Escherichia coli* in later life, compared to non-restricted subjects (51). *F. nucleatum* is specifically associated with genetic and epigenetic defects such as microsatellite instability (MSI) and CpG island methylator phenotype (CIMP), respectively (51). Similarly, consumption of high caloric foods could lead to histone modifications such as methylation and acetylation of the active enhancers, thus augmenting the gene expression pertaining to CRC. Transplantation of colonic microbiota adapted to a high-fat diet into germ-free mice fed on high-caloric diet initiated the reoccurrence of these epigenetic changes (139). In another experiment, human fecal microbiota (from CRC subjects) transplanted to germ-free mice (treated with azoxymethane, CRC model) resulted in increased rate of DNA mutation and decreased DNA methylation involving the gene families of oncogenic *Wnt* and *Notch* pathway (131).

Plant-based derivatives along with microbiome can alter epigenomic changes associated with CRC. Anthocyanins present in freeze-dried black raspberries extracts have shown to induce demethylation of secreted frizzled related protein 2 (SFRP2) promoters, and revive useful probiotics such as *Eubacterium rectale, Faecalibacterium prausnitzii*, and *Lactobacillus* in DSS/AOM colitis-CRC mice model (50). *Sfrp2* hypermethylation and subsequent downregulation are directly associated with development of hepatocellular carcinoma and CRC (140). Impaired gut microbiome activates ten-eleven translocation 3 (TET3) expression in colonocytes which induces demethylation of lamina-associated domains (LADs) leading to epigenetically programmed tumorigenesis and impaired chemotherapeutic response in CRC (141, 142). TET enzymes have active role in inducing demethylation of 5-methylcytosine (5mC) in the CpG islands. TET dioxygenases catalyze conversion of 5mC into oxidized forms, which are further converted to unmethylated cytosine by replication-dependent dilution or base excision repair (141). Resveratrol, a plant based stilbenoid, induces changes in the gut microbiome and is associated with an increased production of butyrate and isobutyrate producing taxa, causing release of anti-inflammatory cytokines (132). This is facilitated by resveratrol-induced inactivation of HDAC, which correlated with upregulation of transcription factor forkhead box P3 (Foxp3). This has several immunomodulatory functions, such as concomitant activation of T-regulatory (T-reg) cells, IL-10 synthesis, and reduction in pro-inflammatory Th1 and Th17m cells. This resulted in inhibition of inflammation in association with restoration of gut microbiome, thereby reducing the risk of colitis-associated CRC (132). *Lactobacillus reuteri* 6475, a commensal and probiotic producing a 2-carbon folate metabolite (5,10-ethenyl-tetrahydrofolyl polyglutamate), biochemically takes part in the transfer of 2 carbon atoms from acetate to homocysteine, resulting in formation of an exclusive amino acid ethionine, instead of conventional methionine. Incorporation of ethionine instead of methionine in proteins leads to decreased methylation and enhanced ethylation of lysine residues in histones (143). Tracking the source of the 2-carbon transporting form of folate by isotope labeling strikingly traces it to acetate in the culture medium of *Lactobacillus reuteri* 6475 (143). Dietary ethionine can result in immunomodulatory effects by suppressing cell mediated immunity and plausibly by NF-κB inhibition (143, 144). However, ethionine also carries carcinogenic potential, which can be reduced by supplementing sufficient methionine (145). This suggests that consumption of certain plant-based extracts and probiotics (such as *Lactobacillus reuteri* 6475) may help to prevent epigenetic alterations associated with CRC.

Finally, the non-coding RNA in the genome also regulates gene expression in CRC (133). Yuan et al. reported 76 differentially expressed microRNAs (miRNAs) in tumor samples, of which 55 were upregulated and 21 were downregulated. miR-182, miR-183, miR-503, and the miR-17~92 clusters were among the most consistently overexpressed miRNA in CRC (146). Genus *Blautia* was inversely correlated with miR-20a, miR-21, miR-96, miR-182, miR-183, and miR-7974, while positively correlated with miR-139, which is significantly expressed in normal tissues (146). However, enrichment analysis has shown that *Akkermansia* is the only genus associated with miRNA, which is linked to CRC pathway (146). This suggests that CRC associated alterations in gut microbiome often changes expression profiles of miRNA linked to cancer pathway. In the case of *F. nucleatum*, selective downregulation of miRNA, such as miR-18a and miR-4802, was shown to activate autophagy, inhibit apoptosis, and induce chemoresistance in HCT116 and HT29 CRC cells (37). miR-18a and miR-4802 post-transcriptionally regulated the expression of pro-autophagic proteins *ULK1* and *ATG7*. However, *F. nucleatum* did not correlate significantly with miR-31 expression, previously shown to be upregulated in CRC with *BRAF* mutation (147, 148). Therefore, *F. nucleatum*-associated CRC plausibly has a key miRNA profile related to its pathogenesis.

The gut microbiome is also an enormous source of lipopolysaccharides (LPS), which flares inflammation and is associated with CRC progression. Exosomal miR-200c-3p notably impedes LPS-induced CRC invasion and migration by targeting zinc finger E-box-binding homeobox-1 (*ZEB-1*) as well as induces apoptosis in HCT116 cells *in vitro* (149). Bacterial small RNA (bsRNA), another microbial genomic component, are 50–400 nucleotides long non-coding RNA molecules which have a central role in regulating post-transcriptional gene expression in various bacterial cellular processes (150). Tarallo et al. using combined metagenomics and small RNA sequencing reported altered human and bsRNA profile in stools of CRC subjects showing alterations in the gut microbiota (151). Based on the bsRNA profile, 15 bacterial species were significantly altered in CRC. *E. coli* bsRNA levels were significantly overrepresented which also correlated with the high abundance of *E. coli* in stool of CRC subjects. *Bacteroides ovatus* bsRNA expression were lower in both CRC and adenomas (151). Stool samples from CRC patients also showed alterations in the gut microbiota, characterized by abundance of *Alistipes putredinis* species and *Firmicutes* phyla. Across human non-coding RNA (ncRNA), miR-378a-3p and piR-11481 were the most differentially expressed miRNA and small ncRNA, respectively. Mjelle et al. reported differential expression of small RNAs from Epstein-Barr virus and *Fusobacterium nucleatum* between colon cancer and adjacent normal mucosa (152). Differential expression of mRNA is also associated with mutational events associated with colon cancer such as microsatellite instability (MSI). microRNA such as miR-335, miR-26 and miR-625 are differentially expressed in association with MSI (152). Thus, it is suggestive that non-coding RNA expression affecting CRC pathogenesis correlates with the composition of the gut microbiome. Moreover, alteration in the expression of human and bacterial small RNA could be a useful tool in the diagnosis of CRC.

Alterations in the gut microbiome pertaining to epigenetic landscape of CRC is highly dependent as well as regulated by our dietary pattern (98). Metabolic conditions like obesity are associated in epigenetic dysregulation and currently emphasis is driven toward identifying the epigenetic markers and the environmental factors deranging them as well as the effect of dietary or therapeutic intervention in regulating these epimutations (153). Paternal high-fat diet consumption leads to beta cell dysfunction in female rat offspring, especially associated with hypomethylation and increased expression of *Il13ra2* (interleukin-13 receptor subunit alpha-2) gene (154). Together with scaffolding protein FAM120A (family with sequence similarity 120A), IL13RA2 activation enhances the survival, invasion, migration, and dissemination of colon cancer (155). Exposure to different dietary constituents, varying pH as well as humid and stable temperature leads to heterogeneity of the oral cavity microbiota, of which certain organisms like *Fusobacterium* are highly implicated in the pathogenesis of CRC (156, 157). *F. nucleatum* consistently increased the expression of miR21 in four colon cancer cell lines potentially *via* theTLR-4/MyD88/NF-κB signaling pathway. Interestingly, elevated levels of miR21 and *F. nucleatum* DNA in the colon tumor tissue was associated with advanced CRC and poor survival (47). miR21 has recognizable role in promoting proliferation of cancer stem cells as well as enhancing angiogenesis while its antagonism reversed the effects (158–160). Dietary fibers are essential source of SCFAs, which can regulate the epigenetic events associated with neoplastic events in human colon cells. Among the SCFAs, butyrate in comparison to acetate and propionate is the most potent metabolite in reducing the proliferation of HCT116 human colon cancer cells by strongly suppressing the cell cycle (G2 phase arrest) and inducing apoptosis (161). Altered gut microbiota in CRC is associated with decline in butyrate producing microbes with corresponding increases in harmful species (162). A prudent mechanism for suppression of CRC by butyrates is by regulating the expression of miRNA (163). *In vitro* experiment by Hu et al. has shown that butyrate's reduced the expression of pro-tumorigenic non-coding RNA such as precursor and mature miR-92a along with primary miR17-92a (164). Nielsen et al. reported that feeding experimental rat with high-amylose potato starch (HAPS), high-amylose maize starch (HAMS), and butyrylated high-amylose maize starch (HAMSB) shifted the gut microbiome toward metabolizing carbohydrates and consequently enhancing butyrate production. HAPS and HAMSB selectively decreased the expression of colonic oncogenic miR17-92 which is protective against CRC (56). This was associated with the downregulation of c-Myc and its overexpression increased the expression of primary miR17-92a. In colon adenoma LT97 cells, butyrate and trichostatin A as histone deacetylase inhibitors downregulated cancer specific miRNAs such as miR-135a, miR-135b, miR-24, miR-106b, and miR-let-7a (165). Butyrate and trichostatin A also upregulated p21 and cyclin D2 expression in non-transfected LT97 cells as compared to cells transfected with miR-106b and miR-135a, respectively. Also, *in vitro* transfection of miR106a mimics into HCT-116 CRC cells decreased the beneficial effect of p21 expression induced by butyrate (166). Thus, consumption of dietary fibers which modulates the gut microbiome to produce metabolites such as SCFAs are beneficial in regulating epigenetic events in CRC.

Overall, it appears that dietary patterns or habits regulating the gut microbiota heavily influences the epigenetic mechanism associated with the pathogenesis of CRC. Further clinical exploration on understanding the outcome of dietary intervention and its modulation of the gut microbiome and the host genome is warranted to validate the therapeutic potential of differential diet-based regimes in management of CRC.

## Analyzing the Composition of the Gut Microbiota and Its Functional Profiling

Methodologically analyzing the gut microbiome and their metabolome is a very complex process and demands the use of advanced techniques to delineate the intricacy related to its composition and function. Culture based methods are the earliest measures to identify the bacterial species based on morphological and biochemical characteristics (167). But culturing underestimates the abundance and diversity of the microbial species in the gut and hence its utility is limited in microbiome research. The smaller 16S ribosome of bacteria contains the ribosomal RNA (rRNA) which is a phylogenetic marker representing the evolutionary divergence of bacteria and consist of several conserved constant regions and species-specific hypervariable regions (V1–V9) (167, 168). Molecular exploration of the hypervariable region is commonly employed to study the complex composition and diversity of the gut microbiome with relative accuracy (167, 168). Its variability at different taxonomic levels can be analyzed to estimate the bacterial genome and its relative abundances (169). These hypervariable regions are distributed between several highly conserved constant regions and variations in the nucleotide sequences within these hypervariable regions reflect evolutionary divergences of bacteria (167). Sequencing these hypervariable regions within the 16S rRNA gene is an essential technique for identification and classification of the bacteria with relative quantification of their abundance. Next generation sequencing is a powerful technique for efficient and cost-effective analysis of the 16S rRNA gene hypervariable regions which could not be effectively mapped using older methods (170). The sequenced data often contain poor quality reads which have to be removed using quality control filters and subsequently based on sequence similarities are clustered into operational taxonomic units or OTU. OTU represents the set of closely matched nucleotide sequences (>97% sequence similarity) which represent either a single or a group of bacterial species (88). The representative sequences from the OTU are mapped in a 16S rRNA gene sequence database for taxonomical classification and determination of diversity, relative abundance as well as annotation related to functional pathways (167). Even though 16S rRNA gene sequencing is a powerful technique in identifying the abundance of different bacterial taxa, it often has certain limitations. 16S rRNA gene sequencing is based on the assumption that >95% similarity is taxonomically associated with genus identification, while >98.7% sequence similarity is useful for distinguishing species (171, 172). But conventionally, the resolution is not enough for intraspecies strain identification, which are characterized by subtle changes in single nucleotide in the gene amplicon. Johnson et al. reported that sequencing the long reads or full-length amplicon of 16S rRNA gene can resolve minor nucleotide changes associated with intraspecies strain identification (173). Moreover, bacterial taxa analyzed by 16S rRNA gene sequencing is based on relative abundance than absolute count of the organism (174). Also, the 16S rRNA reference database is incomplete and does not cover all the reference sequences pertaining to specific microbial species from the diverse gut microbiota (167, 174). Amplification of erroneous sequences termed as chimera, which consist of a single amplicon originating from two different sources during the polymerase chain reaction (PCR) cycles could lead to wrong species identification and annotations (174). These drawbacks associated with inaccurate identification of bacterial species, incomplete 16S rRNA gene reference database and sequencing errors are major pitfalls associated with 16S rRNA gene sequencing and demand newer techniques to overcome these inadequacies and improve the resolution in identifying bacterial taxa to subspecies or strain levels. Shotgun metagenomic analysis using high throughput sequencing is another powerful technique, which collectively analyzes all the genes and genomes of the microbiota in the given specimen and is the next step for discretizing higher level of bacterial taxonomical characterization to achieve higher degree of sequence similarity and subspecies identification (167). This method circumvents the traditional 16S rRNA gene sequencing as well as provides better information on the functional capabilities of the microbiome (167). In this method the complete metagenome (cumulative genome of bacteria and host) obtained from the sample is fragmented into short segments and further sequenced using high throughput sequencing. Adequate filters are required for rarefaction of bacterial genomic component and remove the contaminants from host genome. The genome data is further analyzed using bioinformatic pipelines to delineate bacterial taxa. Compared to 16S rRNA gene sequencing, metagenomics reveals more information on low abundance genera and is more useful in determining microbial diversity when sufficient reads are available (175). Nanopore sequencing, using workflow, called Lathe (tool for generating bacterial genome from metagenomic data based on nanopore long read sequencing) could be used to assemble closed circular genome by incorporating long read assembly, short read error correction, and genome circularization (176, 177). It enhances binning assembly from assembled contigs as contiguity from short reads is often contaminated by repeat elements. This determines high-quality contiguous and circular bacterial genomes from diverse human gut microbiome. However, sequencing the closed circular genome from long reads requires extraction of high molecular weight DNA extraction which is technically challenging due to its low yield.

Newer modalities like metatranscriptomics, metaproteomics, and metabolomics together aid in understanding the functional role of the human gut microbiome (178, 179). Metatranscriptomics, which is based on RNA-seq further takes the bacterial taxonomical classification to the next level by determining the expressional significance of the proteins associated with altered gut microbiome (167). Longitudinally pairing metagenomics with metatranscriptomics will holistically elucidate the functional dynamics of the gut microbiome in specific diseases (180). Furthermore, metaproteomics and metabolomics unravels the functional significance of all expressed proteins and generated metabolites by the transforming gut microbiota and provides key information on its predictive or definitive causal relationship in different human diseases (181–183). Metaproteomics involves the study of the complete proteins produced by the gut microbiota in the provided specimen, which elucidates the functional aspects of microbial gene expression (167). Correspondingly, metabolomics deal with the complete metabolite profiling of the given specimen. The three useful and pivotal techniques for studying the metabolomic profile of the stool specimen commonly includes gas chromatography-mass spectrometry (GC–MS), liquid chromatography-mass spectrometry (LC-MS), and capillary electrophoresis-mass spectrometry (CE-MS) (181, 184). Metabolomic workflow involves sampling, sample processing, instrumental separation, metabolomic data analysis, and interpretation (181). Metabolomic pipelines can further characterize the metabolomic data in relation to gut microbiota composition. Recently, combined use of comprehensive metabolomic dataset and random forest-based machine learning algorithm have unraveled novel metabolic and biochemic pathways characterized to *Bacteroides* genus (185). This is a new milestone in the area of metabolomics as machine learning can incorporate technological edge in determining the metabolomic changes pertaining to specific microbe. Natural dietary components such as polyphenols are effective antioxidants and prebiotics which are processed by the gut microbiota into metabolites with immunomodulatory functions. A high throughput analysis known as foodomics utilizes various omics-based approach to identify suitable active polyphenols in the natural food source (186). This approach can also delineate the possible health benefits of consumption of dietary nutrients such as polyphenols. Microbiomics, nutri(epi)genomics, and metabolomics, collectively are high throughput “omics” technologies which give better understanding on the vital role of food bioactive substances in human health in a personalized manner, considering the interindividual differences in their metabolism, bioavailability, and bioefficiency (187). Overall, it can be summarized that omics-based approaches have pivotal role in understanding the human gut microbiota and the functional significance of its gene expression.

A solitary metagenomics analysis may be inadequate to establish association between the host, gut microbiome, and metabolite axis. Meta-analysis of metagenomics data is a powerful method for summarizing the impact of gut microbiota and its metabolites in CRC across different studies. Wirbel et al. in a meta-analysis based on eight fecal shotgun metagenomics studies (total 768 fecal metagenomes) identified potential microbial markers and altered metabolites associated with CRC (188). The analysis of diverse CRC metagenomes revealed a key set of 29 microbial markers, which were significantly associated with CRC. These also included genera such as *Fusobacterium, Porphyromonas, Parvimonas, Peptostreptococcus, Gemella, Prevotella*, and *Solobacterium*, which were previously associated with CRC (188). Functional analysis of the CRC metagenomes showed an upregulation of pathways related to protein, glycoprotein, and organic acid metabolism, while carbohydrate metabolizing genes were depleted (188). This suggested that the healthy gut microbiome which preferentially metabolized carbohydrate shifted toward an amino acid utilizing pattern in CRC. Interestingly, Thomas et al. in another meta-analysis involving large-scale CRC metagenomic datasets observed greater microbial richness in the gut microbiome of CRC subjects when compared to controls (189). This pattern was contributed by the overabundance of microbial species from the oral cavity in the gut microbiome of CRC subjects. CRC biomarker species such as *F. nucleatum, Solobacterium moorei, Porphyromonas asaccharolytica, Parvimonas micra, Peptostreptococcus stomatis*, and *Parvimonas* spp. were commonly observed in most of the individual studies. Analysis of the fecal metabolic profiles from the datasets show an abundance of the gene choline trimethylamine-lyase, which is required for the bacterial synthesis of trimethylamine, a potent carcinogen associated with CRC (189, 190). Thomas et al. also reported that predicting CRC based on an independent metagenomic dataset would incur errors and therefore performed alternative analysis based on leave one dataset out (LODO) predictive model which can be potentially useful in clinical scenario (189). Thus, meta-analysis-based approaches for comparing different metagenomic studies could be an effective method for identifying novel CRC associated microbial biomarker species and metabolites from large scale datasets.

## Conclusion

Emerging evidence suggests a significant association between the gut microbiome and colorectal cancer. As a result, dietary constituents such as dietary fibers, phytomolecules, n-3 PUFAs, prebiotics, probiotics, and postbiotics may offer benefits in the prevention of CRC through favorable alterations in the gut microbiome. More specifically, dietary and lifestyle factors may enrich the growth of healthy microbes and suppress the non-beneficial strains. Beneficial strains of gut microbiome produce enterocyte-friendly metabolites, such as SCFAs, and may protect the mucosa against inflammation and induction of oncogenic pathways. At this time, prospective data examining this anti-cancer approach is lacking. Future studies should examine the microbiome impact of dietary risk factor modification in patients at high-risk for CRC.

## Author Contributions

RK and SA: conceptualization. MR: methodology. VR, PM, and RT: literature search. SA: writing—original draft preparation. MR, VR, AS, RT, PM, and AK: writing—review and editing. All authors have read and agreed to the published version of the manuscript.

## Conflict of Interest

The authors declare that the research was conducted in the absence of any commercial or financial relationships that could be construed as a potential conflict of interest.

## Publisher's Note

All claims expressed in this article are solely those of the authors and do not necessarily represent those of their affiliated organizations, or those of the publisher, the editors and the reviewers. Any product that may be evaluated in this article, or claim that may be made by its manufacturer, is not guaranteed or endorsed by the publisher.
